# Eye movements efficiently expose single cone photoreceptors to global scene color statistics

**DOI:** 10.1016/j.isci.2026.114948

**Published:** 2026-02-09

**Authors:** Takuma Morimoto, Luna Wang, Kinjiro Amano, David H. Foster, Sérgio M.C. Nascimento

**Affiliations:** 1 Physics Center of Minho and Porto Universities (CF-UM-UP), Braga, Portugal; 2 Department of Experimental Psychology, University of Oxford, Oxford, UK; 3 Department of Psychology, University of Edinburgh, Edinburgh, Scotland; 4 Department of Electrical and Electronic Engineering, University of Manchester, Manchester, UK

**Keywords:** Biological sciences, Physiology, Biophysics

## Abstract

Our visual experience is a dynamic consequence of our actions, most notably our continuously shifting gaze. These shifts directly influence the spectral input received by individual cone photoreceptors. The present study tested how gaze shifts shape the chromatic diet of single cones and their relationship to global adaptation. Eye movements were recorded from observers freely viewing natural scenes outdoors and from observers freely viewing hyperspectral images of the same scenes indoors on a computer-controlled laboratory display. From the hyperspectral data, spatially local histograms of excitations in long-, medium-, and short-wavelength-sensitive cones were accumulated over time. A simulated global illuminant change was then introduced into the images to test how well local retinal adaptation might discount its effects. Despite the highly non-uniform pattern of natural gaze behavior, the results suggested that individual cones may experience the global color statistics of full scenes within the first several seconds of viewing. This effective sampling could support robust adaptation, allowing local adaptation mechanisms to compensate for illumination changes.

## Highlights

• Natural gaze makes single-cone chromatic diets converge to scene-wide statistics
• Local vs. global L/M/S cone histograms correlate strongly within ∼10 s of viewing
• Local von Kries scaling from cone samples nearly matches global adaptation
• Random-walk sampling approximates observers, but chromatic-salience underperforms

## Graphical abstract

**Figure:**
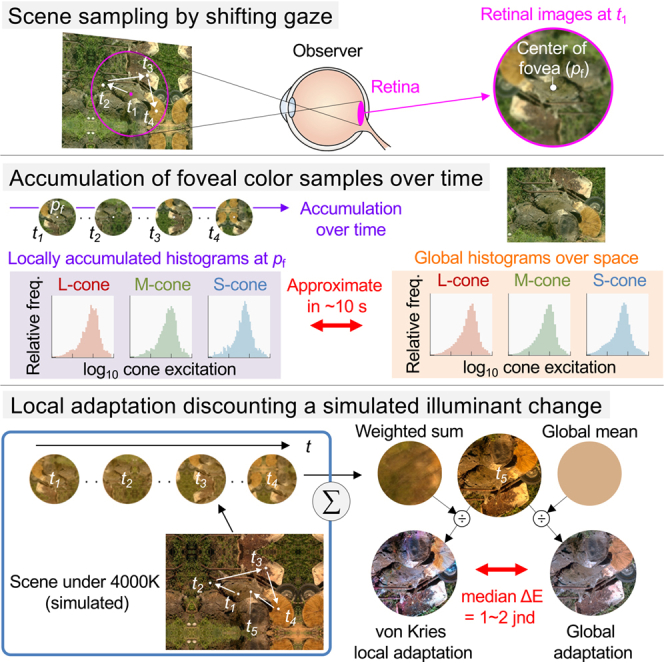

## Introduction

Characterizing our everyday exposure to natural optical environments may offer insights into the role of individual visual mechanisms. These mechanisms need to deal with the complex, varying patterns of spectral radiation incident at the eye,^1^^,^^2^^,^^3^ sometimes referred to as our “spectral diet.”.^4^ Yet quantifying the signals received by individual cone photoreceptors presents a challenge: our spectral diet is not a passive reflection of a static external world, but is dynamically shaped by our active behaviors, most importantly our shifting gaze. Although there have been efforts to characterize physical regularities in natural scenes and their connection to a range of visual functions^5^^,^^6^^,^^7^^,^^8^^,^^9^^,^^10^^,^^11^^,^^12^^,^^13^ and how our sampling behavior interacts with experienced image statistics,^14^^,^^15^^,^^16^^,^^17^^,^^18^^,^^19^^,^^20^ the efficiency of this sampling remains unclear.

At any given moment, alongside the dynamics of our behavior, the complex spatial structures of natural environments^21^^,^^22^ provide different physical inputs to cones at different retinal positions. This suggests that our cones, distributed over the retina, receive markedly different physical inputs from moment to moment, potentially affecting visual function across the visual field. In some species, there are structural consequences. An extreme example is the visual field of mice, where only the upper visual field appears to support color discrimination,^23^ presumably in response to the asymmetry between mouse views of the ground and the sky.^24^ In contrast, our more flexible patterns of behavior allow us to compensate for the spatial inconsistency of photoreceptor input by shifting our gaze. But whether these shifts ensure each cone experiences a variety of spectra more representative of the entire scene is uncertain. *A priori*, there is no reason to expect the highly uneven and often unpredictable distribution of natural gaze, whose priority may be more to explore the salient content of a scene,^25^^,^^26^ should deliver approximate adaptational uniformity over the whole scene. The first goal of this study was to determine whether our natural sampling behaviors could allow individual cones to access scene color statistics or are limited to their local environment, with consequences for visual detection, discrimination, and interpretation of scene contents.^20^^,^^25^^,^^27^

These sampling behaviors are particularly important in color vision because retinal adaptation is thought to be one of the main mechanisms for color constancy,^28^^,^^29^^,^^30^^,^^31^ a visual ability to perceive object or surface color consistently despite changes in the spectrum of the illumination. The required adaptation is routinely assumed to be achieved by scaling each class of cone signals across the whole retina by a single multiplicative factor.^32^ This operation can be represented mathematically by a diagonal matrix transformation,^32^ which has been the basis of many computational color constancy algorithms^33^ and color appearance models, standardized by the Commission Internationale de l'Eclairage (CIE) in protocols such as CIECAM02 and CIECAM16.^34^^,^^35^ Yet this scaling was originally proposed by von Kries as a local, not global, mechanism.^28^^,^^36^ Local scaling may be more plausible at the photoreceptor level, but its efficacy seems not to have been empirically tested. The second goal of the present study was to determine whether local retinal adaptation adequately approximates the effects of global retinal adaptation.

The approach was necessarily computational, given the experimental difficulties of distinguishing between local and global adaptation effects during observers’ free viewing of natural scenes. No assumptions were made about the nature of post-receptor processing, other than being the same whether stimuli were derived from gaze distributions decided by salience or by uniform sampling over a scene. Moreover, because of the nonuniformity of natural gaze distributions, neither goal can be addressed by simply taking a moving subset of scene spectral data. Rather, both observer gaze data and spectral radiance data are needed at each point in a scene to compute the spatial pattern of cone excitations across a retina, along with control data from three specific alternative kinds of gaze behavior, one following uniform random sampling, another following a random walk and a third restricted to viewing only a chromatically salient region (i.e., the region with maximum chroma) within the scene. Although gaze data with RGB images are widely available,^37^^,^^38^^,^^39^^,^^40^ gaze data with hyperspectral images, which provide a continuous radiance or reflectance signal at each pixel of an image, have been less accessible.

In each of seven outdoor environments, a hyperspectral radiance image of the scene was first acquired from the observer’s eye position, and then gaze patterns were recorded from two of the authors freely viewing the scene for 5 min. Additional gaze data were collected in the laboratory, where five observers (including the two authors) freely viewed the scene images presented at the same visual angle on a color monitor. In computational simulations with these gaze data, the history of individual cone excitations in the fovea was then estimated from the hyperspectral images under natural daylight illumination, with an average correlated color temperature of 5,571 K computed across scenes. The natural illumination was then replaced by a simulated global illuminant with correlated color temperature 4,000 K, characteristic of the setting sun, to test whether local retinal adaptation, based on accumulated foveal cone signals, could discount the effects of the simulated global change in illumination spectrum. The results suggest that even over short periods, individual cones tend to experience the statistics of full scenes, despite our non-uniform gaze behavior, with local adaptation potentially compensating for illumination changes nearly as well as global adaptation. This compensation may help to maintain our stable local perception of scene color despite changes in scene illumination.

## Results

Results for observers’ gaze behaviors are presented first for measurements with five observers indoors and then with two observers outdoors.

### Gaze shifts in each scene

**Figure 1:**
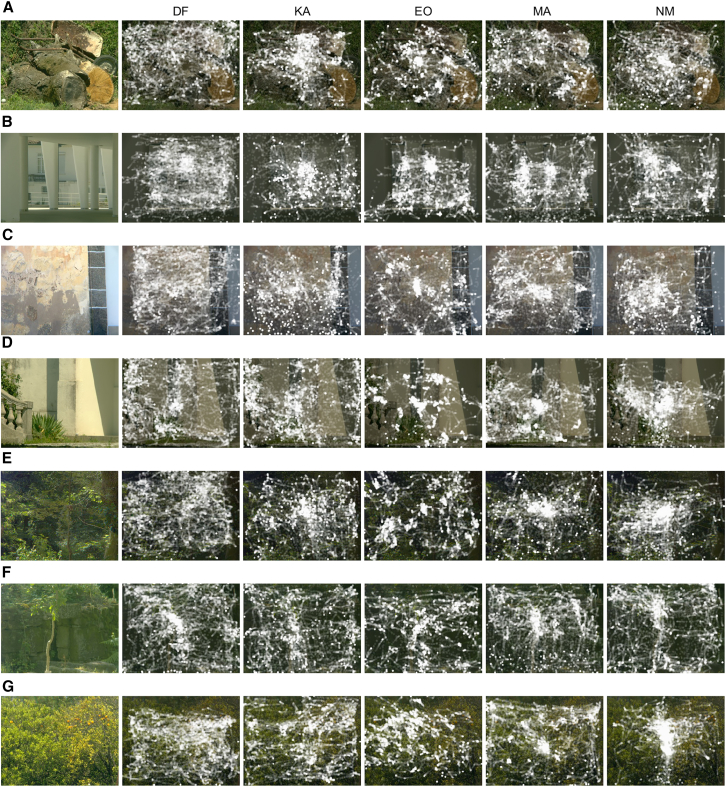
Natural scene images and gaze measurements in indoor enviornments. Images of seven natural scenes (A)-(G) are shown, with the corresponding gaze patterns recorded indoors for five observers displayed to the right of each scene. The gaze data are superimposed on the darkened original image.

Figure 1 shows on the left images of the scenes and to the right the corresponding gaze patterns (indicated by white dots) recorded indoors with five observers. As expected, gaze distributions were markedly nonuniform, even in scenes that had relatively fine-grained variation, as in Figure 1G. The individual differences in gaze behavior evident here are well known in real-world scene viewing.^41^

**Figure 2:**
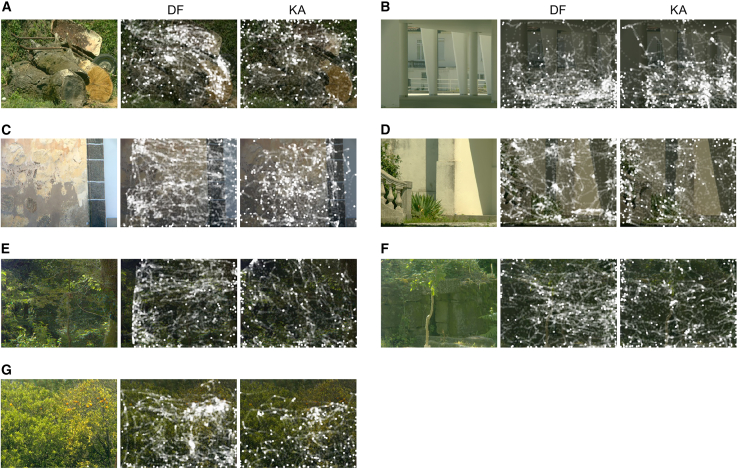
Images of seven natural scenes, as in Figure 1, and, to the right of each, gaze patterns recorded outdoors for two observers

Figure 2 shows the gaze patterns recorded outdoors for observers DF and KA. Some gaze distributions differed from their indoor counterparts, and others were similar. The important measure, though, is the potential relationship between cumulative local adaptation and global adaptation, which is addressed in the following section.

**Figure 3:**
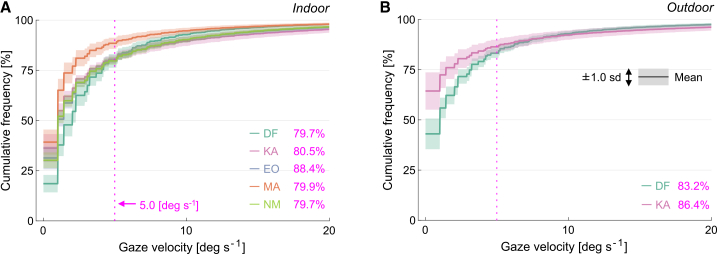
Gaze velocity distributions in indoor and outdoor environments Cumulative histogram of gaze velocity for (A) indoor environments (five observers) and (B) outdoor environments (two observers). Values were averaged across seven scenes. The curved line represents the mean, and the shaded region indicates ±1 standard deviation across the scenes. The percentages denote the cumulative frequency for each of the five observers up to 5.0 deg s^−1^, indicated by the vertical dotted line. The maximum speed was 387 deg s^−1^ for the indoor environment and 403 deg s^−1^ for the outdoor environment.

Notwithstanding the individual differences in the distributions, gaze-shift velocities were consistent across participants both indoors and outdoors. Figure 3 presents cumulative histograms of these velocities. Approximately 80% or more of the gaze shifts recorded had velocities below 5 deg s^−1^, indicating that most gaze shifts were slow and continuous. Since higher velocities may have introduced temporal frequencies that exceeded the cone pathway response, gaze shifts greater than 5 deg s^−1^ were excluded from subsequent analyses. This threshold is conservative, as saccades are typically defined by higher velocities (e.g., > 15 deg s^−1^,^42^ or > 26.8 deg s^−1^^43^).

### Correlation between sampling by individual cones at the fovea and the global cone response across a scene

**Figure 4:**
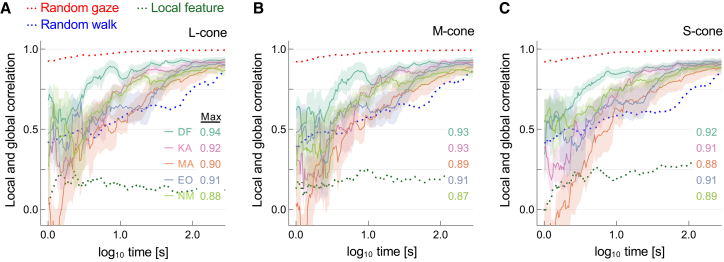
Correlation between local and global cone responses over time for indoor gaze recordings from five observers The three panels display the Pearson product-moment correlation coefficients between local histograms constructed from the history of cone excitation at the fixation point and global histograms derived from cone excitation across the entire image, for each cone class: (A) L-cone, (B) M-cone, and (C) S-cone. These correlations are averaged across seven scenes and plotted against the base-10 logarithm of time since gaze onset. Curve colors indicate observers, and shaded regions denote ±1 standard error (SE) across scenes. Dotted red, blue, and green curves represent predictions from the random gaze, random walk, and local feature models, respectively.

Figure 4 summarizes the time course of the correlations between local cone activity at the gaze center (i.e., fixation point) and global cone response derived from the entire image in indoor gaze recordings. The vertical axes show Pearson product-moment correlation coefficients between local and global histograms of L, M, and S cone excitations, characterizing the spectral diet of cones over time, plotted against time for each of the five observers. The extent to which samples from individual cones capture global scene statistics should be evident in an increase in the correlation coefficient between local and global histograms with time from gaze onset. For all observers, the correlation increased rapidly during the first 10 s and gradually thereafter. The maximum correlation ranged from 0.87 to 0.94. The trend was similar across cone classes, indicating that equating adaptation state by gaze shifts was not specific to a single cone type.

Predictions from control data obtained with uniform random sampling provided an upper limit on what gaze behavior could achieve if required solely to approximate the effects of global adaptation. Unsurprisingly, the correlation approached its asymptote within the first few seconds. Predictions from control data based on a random walk model of sampling behavior were more relevant to human gaze behaviors, presumably because, unlike random gaze, locations change progressively, approximating natural gaze behavior. In contrast, a local-feature model constrained to fixate only around the region of maximum chroma showed lower correlations with global statistics than human observers, underscoring the importance of natural gaze shifts for effectively sampling scene information.

**Figure 5:**
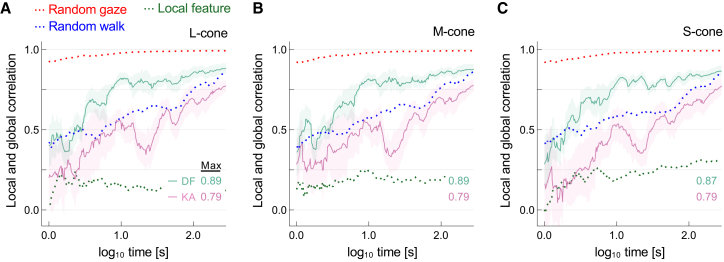
Correlation between local and global cone responses over time for outdoor gaze recordings from two observers (DF and KA) Three panels show correaltions for (A) L-cone, (B) M-cone, and (C) S-cone responses. All other details are also the same as in Figure 4.

Figure 5 shows the corresponding correlations for the outdoor gaze recordings with the two observers who also participated in the indoor measurements. The correlation increased over time in a similar way across cone classes. For observer DF, it increased rapidly during the first 10 s and gradually reached a maximum value between 0.87 and 0.89. For observer KA, the increase was more gradual, with a dip before eventually peaking at around 0.77–0.78. As noted earlier, individual gaze behavior varies in real-world scene viewing,^41^ but even with the additional uncertainties of outdoor recording, most of the approximation to global adaptation appears to take place within the first 10 s of viewing.

In the foregoing correlation analysis, gaze shifts faster 5 deg s^−1^ were excluded. To test the robustness of our findings, we repeated the analysis without the exclusion, assuming that fast eye movements also contributed to the visual diet. The results remained virtually unchanged, with no notable deviation from the main findings.

### Local retinal adaptation and von Kries’ scaling

Assessing how well cumulative local retinal adaptation approximates global adaptation needs a way of comparing their effects on image color appearance. To that end, changes in appearance were quantified by mapping each image into the approximately uniform color space CIECAM16-UCS^44^ and taking color differences Δ*E* at each point on the retinal image. Details are given in Methods (Analysis of local retinal adaptation and influence of illumination).

**Figure 6:**
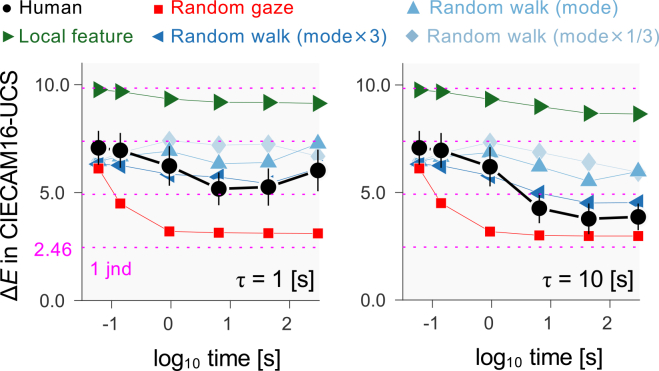
Discounting illumination color by local retinal adaptation over time The left and right plots show time constants (τ) of 1 s and 10 s, respectively. Median CIECAM16-UCS color differences Δ*E* between images corrected by local and global diagonal matrix transformations are plotted against the logarithm of the time from gaze onset. Data are averaged across two observers and seven scenes, with simulations based on random gaze, random walk, and local features. The horizontal dotted lines show increments in multiples of 1 jnd. Error bars show ±1 standard error (SE) across scenes.

Figure 6 plots the median Δ*E* between images where the influence of 4,000 K global illumination was corrected using local and global diagonal matrix transformations, illustrating how closely local chromatic adaptation approximates global chromatic adaptation. The two panels show results for different adaptation time constants.

To provide a reference point for assessing these color differences, Δ*E*, a threshold value or just noticeable difference (jnd) was derived from earlier work with images of the kind used here,^45^ which suggested a jnd of about 2.2 in CIELAB space.^34^ This CIELAB jnd was transformed to a more relevant CIECAM16-UCS jnd of 2.46 in a way similar to that used previously to transform jnd values between color spaces.^46^

As expected, the color differences in Figure 6 were slightly smaller for a time constant of 10 s than for 1 s. To confirm this statistically, minimum Δ*E* values were calculated for each of the 7 scenes at both time constants of adaptation (1 s and 10 s). A one-way repeated measures ANOVA revealed a significant effect of time constant (*F*(1,6) = 22, *p* < 0.01) on the minimum Δ*E* averaged across scenes. To quantify the impact of introducing a 4,000 K global illuminant to a scene, we computed Δ*E* values without applying illuminant correction. The average Δ*E* across scenes was 29, highlighting the substantial color shifts introduced by the illuminant. Against this value, the local diagonal matrix transformation based on the first three image frames (the leftmost data point) brought marked benefit, with Δ*E* values decreasing progressively to a minimum around the three end time points (6.4 s, 44 s, and 300 s). Note that even with a shorter time constant of 1 s, the lowest Δ*E* was 5.2, about 2 jnd values.

For scenes where pixel chromaticities showed little spatial variation, and the mean chromaticity was close to gray (e.g., scenes in panels (B) and (C) in Figure 1), Δ*E* values were small at the first frame but then decreased more slowly afterward, showing that the benefits of gaze shifts depend on chromatic variation. As before, the random gaze model and the local feature model provided a much poorer fit than the random walk model. It is worth noting that the *mode × 3* model more closely resembled human observers than the *mode × 1* or *mode × 1/3* models. In other words, human gaze patterns were more efficient from an adaptation perspective than random movements with the same most frequent speed (mode), and this efficiency was comparable to that of faster random movements.

**Figure 7:**
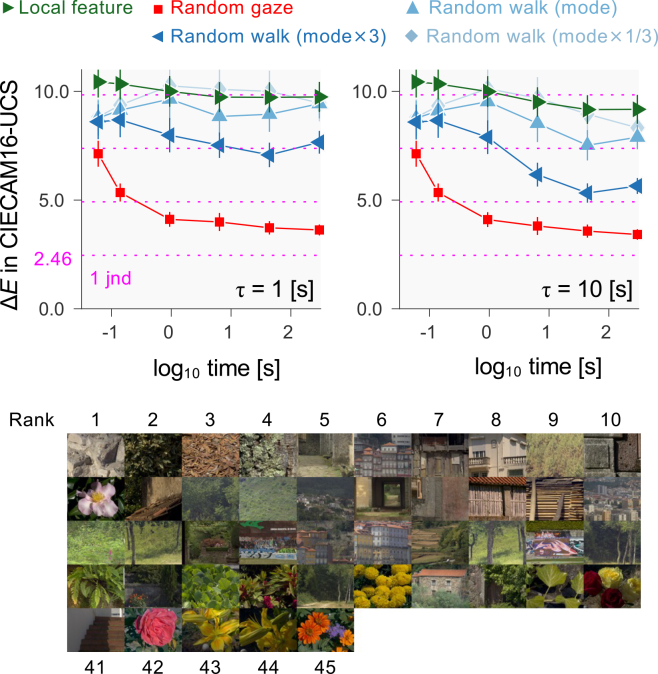
Simulations with local feature, random gaze, and random walk models averaged across the 45 scenes in the montage Images are shown in ascending order of minimum Δ*E* for the random walk model (three-times mode). The horizontal dotted line shows increments in multiples of 1 jnd. Error bars show ±1 standard error (SE) across scenes.

The closeness of the random walk model to observer performance allows a test of the generalizability of the present findings from seven natural scenes to larger samples, namely the additional 45 images shown in Figure 7. The time courses of the color differences Δ*E* are similar to those in Figure 6.

In summary, it seems that gaze shifts can indeed largely discount the adaptational effects of global illumination changes, especially over longer time periods.^47^

## Discussion

We routinely encounter a variety of natural optical environments, often with complex structures. How single cones might accommodate this changing spectral diet during natural gaze behavior was addressed here by analyzing gaze patterns in relation to local and global cone activity accumulated over time. Two main mechanisms were identified. One was the convergence over time between the physical signals experienced by individual long-, medium-, and short-wavelength-sensitive cones and the global color statistics at the fovea. This implies that our active sampling behavior largely standardizes the spectral diet of individual cones. The other mechanism was the convergence over time between the effects of local adaptation in discounting illumination changes and global adaptation based on average scene color.

### Adaptation to visual environments

As noted earlier, visual adaptation can occur over multiple time scales,^48^^,^^49^^,^^50^^,^^51^^,^^52^^,^^53^ and a variety of studies have demonstrated the normalizing effects of chromatic adaptation. These effects include eliminating color differences between the fovea and periphery by adapting to macular filtering^54^; changes in color perception after wearing colored glasses^55^; violet-blue color shifts diminishing after cataract surgery^56^; and seasonal biases in wavelength settings of unique yellow.^57^ The present analysis suggests that adaptation by gaze shifts can be added to this resource of normalizing mechanisms.

The effectiveness of adaptation by gaze shifts is, though, contingent on the kinds of gaze behavior found in practice. The most common rationale for that behavior has been bottom-up salience,^25^^,^^58^ that is, the presence typically of local features such as line orientation and color but also objects,^59^^,^^60^ which by virtue of their distinctiveness attract an observer’s attention. It seems that their distribution is sufficient for gaze behavior to capture much of global color statistics, albeit less efficiently than uniform random sampling.

### Implications for color constancy

Although our spectral diet is known to play an important role in color appearance and color constancy,^5^^,^^61^ the relationship between local and global mechanisms has received less attention.^28^ The sampling of a broader extent of the environment identified here is crucial. Without the spatial averaging of adaptation delivered by eye movements, shadowed regions in a scene under local adaptation might look more like directly illuminated regions. In the limit, where adaptation is entirely local, the distinction between surfaces would be lost, an effect analogous to the extra-foveal fading experienced in Troxler’s effect.^62^

Not all color constancy phenomena depend on global adaptation. When color constancy is assessed operationally,^63^ by asking observers to distinguish illuminant changes from material changes, they can make the required judgments rapidly and accurately. This kind of constancy, referred to as immediate color constancy^64^ or sometimes instantaneous color constancy,^65^ is thought to be underpinned by inferences about the color relations between surfaces,^66^ which are largely independent of the illumination.

### Limitations of the study

This study had several technical constraints. First, the temporal resolution of the eye tracker was limited, hindering the analysis of rapid gaze shifts, including fixational eye movements,^27^ but leaving broadly intact the statistical sampling of scenes. Second, conducting outdoor measurements presented significant challenges, including changes in the lighting environment over time, which limited the number of outdoor recordings that could be used in the analysis. Even so, the time course of performance was similar to that recorded indoors. Third, gaze data were gathered for a free-viewing task because of the difficulties with implementing a random target search task outdoors. The nature of the task and visual attention are both known to influence gaze behavior.^67^^,^^68^^,^^69^ Coincidentally, free viewing may have contributed to the success of the random-walk model in approximating observer responses, given its indifference to scene content. Fourth, data from gaze recordings were averaged over scenes, since there were too few for individual analysis, and did not fully represent the scope of real-world scenes. Additionally, most had little or no sky, which, by contrast with the ground, would have introduced large chromatic and luminance transients across the retina on direct viewing.^70^ Finally, although a simple decaying exponential model of adaptation sufficed, it could have been extended to include higher-order temporal filtering,^71^ which could dynamically reshape the adaptation state over time, together with photoreceptor noise^72^ and the detailed variation in the retinal cone mosaic.^73^^,^^74^ Nonlinearities in the adaptation process and spatial dependencies in post-receptoral pathways, such as pooling or averaging of neighboring cone signals, might further modulate the balance between local and global adaptation and smooth retinal responses.

Even with these limitations, the central aim of this work was to characterize the physical input signals to cones under natural viewing conditions. Within this scope, the study offers insights into how the dynamics of real-world gaze behavior shape the temporal course of chromatic adaptation.

## Resource availability

### Lead contact

Further information and requests for resources and reagents should be directed to and will be fulfilled by the Lead Contact, Takuma Morimoto (takuma.morimoto@psy.ox.ac.uk).

### Materials availability

This study generated no new materials.

### Data and code availability

• Gaze data for all observers and seven hyperspectral images used in the main analysis are available on GitHub https://github.com/takuma929/gaze_sampling_color_statistics.git.
• All original codes required to reproduce the figures in this article are available on GitHub: https://github.com/takuma929/gaze_sampling_color_statistics.
• Any additional information required to reanalyze the data reported in this article is available from the lead contact upon request.

## Acknowledgments

TM was supported by a Sir Henry Wellcome Postdoctoral Fellowship from 10.13039/100010269Wellcome Trust (218657/Z/19/Z). This work was supported by the Portuguese Foundation for Science and Technology (FCT) in the framework of the Strategic Funding UID/04650/2025 (https://doi.org/10.54499/UID/04650/2025), the 10.13039/501100000275Leverhulme Trust (RPG-2022-266), and the 10.13039/501100000266EPSRC (EP/W033968/1). For the purpose of open access, the author has applied a CC BY public copyright license to any Author Accepted Manuscript version arising from this submission.

## Author contributions

Conceptualization: T.M.; data curation: T.M., K.A., and S.M.C.N.; formal analysis: T.M.; funding acquisition: T.M., S.M.C.N., and D.H.F.; investigation: T.M., K.A., S.M.C.N., and D.H.F.; methodology: T.M., K.A., and S.M.C.N.; project administration: T.M., K.A., and S.M.C.N.; resources: T.M. and S.M.C.N.; software: T.M. and S.M.C.N.; supervision: S.M.C.N.; validation: T.M.; visualization: T.M.; writing – original draft preparation: T.M.; writing – review and editing: T.M., L.W., K.A., S.M.C.N., and D.H.F.

## Declaration of interests

The authors declare no competing interests.

## STAR★Methods

### Key resources table

**Table:** 

REAGENT or RESOURCE	SOURCE	IDENTIFIER
**Deposited data**		

7 hyperspectral radiance images	This study	https://github.com/takuma929/gaze_sampling_color_statistics.git
Gaze data	This study	https://github.com/takuma929/gaze_sampling_color_statistics.git

**Software and algorithms**		

MATLAB 2024b	MathWorks	https://uk.mathworks.com/products/new_products/release2024b.html
50 hyperspectral radiance images	Foster et al. (2022)^48^	https://doi.org/10.48420/14877285
Human cone pigment spectral sensitivities	Stockman et al.^75^ ; Stockman andd Sharpe^76^	http://cvrl.ioo.ucl.ac.uk/

### Experimental model and study participant details

#### Model (simulated observers)

To compare with human gaze behavior, we simulated three model observers. The first used random gaze, in which gaze positions were selected at random in each frame. The second used a random-walk strategy, where gaze positions resulted from random shifts in direction with a fixed step size.^77^^,^^78^ The third model looked at only a chromatically salient local region in a given scene, defined as the 5 × 5 pixel region with the highest mean chroma value (*C*∗ in *L*∗*a*∗*b*∗ color space), and performed a local random walk within a 0.5-deg-diameter area centered on that region. For all models, gaze positions were constrained to remain within the image boundaries. Step size for the random walk model was based on recorded angular velocities, with values equal to the mode (2.3 deg s^−1^) and one-third and three times the mode (0.77 deg s^−1^ and 6.9 deg s^−1^). The step size for the local-feature model was set to one-third of the mode (0.77 deg s^−1^). Gaze positions of these simulated observers were calculated for 5 min at the same temporal frequency as with the eye tracker, resulting in 15,000 frames. The gaze position with the first frame was set to the same random value for both models and their variations. Data were analyzed in the same way as with human gaze.

#### Observers

For outdoor scene measurements, the observers were authors KA and DF, both male, aged 33 and 59 years, respectively. For indoor measurements, these observers were joined by three others EO, MA and NM, also male, aged 24–34 years. All observers had normal or corrected-to-normal visual acuity and normal color vision as assessed with an anomaloscope (Oculus HMC Anomaloscope, Oculus, Wetzlar, Germany).

### Method details

#### Gaze tracking

Gaze patterns were recorded from the observer’s right eye with an infrared eye tracker system (iView, version 3.01, SensoMotoric Instruments, Germany), attached to a chin and forehead rest anchored to a table, as illustrated in Figure S1A. Eye position was estimated from the corneal reflection. The system had a sampling rate of 50 Hz (i.e., data interval 20 ms) and the tracking area in the frontoparallel plane subtended a visual angle 30° horizontally and 25° vertically. Measurements outdoors were made under a parasol to prevent direct light from reaching the eye tracker (Figure S1B). For the indoor measurement (Figure S1C), observers were positioned in front of a CRT monitor (GDM-F400T9, 19-inches, 1600 × 1200 pixels, Sony, Tokyo, Japan) with the eye tracker system. Scene images were displayed on the monitor with the same visual angle as the outdoor measurements, through a graphics card (VSG 5, Cambridge Research Systems, Rochester, UK) that allowed 8-bit depth per each RGB phosphor. The range of pixels outside of the monitor’s chromaticity gamut varied from 0.0% to 6.9% across 7 scenes. Overall luminance levels were reduced to fit the luminance range allowed by the monitor, and the mean luminance level ranged between 8 and 15 cd/m^2^.

For calibration of the eye tracker outdoors, 3 × 3 gray fixation targets were placed in the scene to form an approximately regular array from the observer’s viewpoint. A grayscale image with the fixation marks in place (Figure S1D) was loaded into the eye-movement software. The observer looked at each mark in turn during the calibration procedure. For the indoor measurement, a 3 × 3 calibration grid was embedded at the same image locations as in the outdoor measurement, with the procedure otherwise identical to that used outdoors.

#### Hyperspectral imaging

The seven hyperspectral radiance images used in the fixation analysis were acquired as described previously,^79^^,^^80^ with the hyperspectral camera placed approximately at the observer’s viewpoint. In brief, the hyperspectral imaging system was based on a low-noise Peltier-cooled digital camera (Hamamatsu, model C4742-95-12 ER, Hamamatsu Photonics K. K., Japan) with a fast tunable liquid-crystal filter (VariSpec, model VS-VIS2-10-HC-35-SQ, Cambridge Research & Instrumentation, Inc., Massachusetts) mounted in front of the lens whose peak-transmission wavelength could be varied continuously. Immediately after acquisition, the spectrum of light reflected from a small neutral (Munsell N5 or N7) reference surface in the scene was recorded with a telespectroradiometer (SpectraColorimeter, PR-650, Photo Research Inc., Chatsworth, California). Raw images were corrected for dark noise, spatial nonuniformities, stray light, and any wavelength-dependent variations in magnification or translation. Where necessary for computational purposes, radiance images were converted to effective reflectance images by pixelwise division by the estimated spectrum of the direct illumination recorded from the reference surface.^81^ Color images derived from the hyperspectral images by are shown elsewhere. To supplement the main analysis, an additional 45 hyperspectral reflectance images were also used.^46^

Each hyperspectral image had dimensions approximately 1344 × 1024 pixels, corresponding to a camera angle of approximately 6.9 ° × 5.3 °, and spectral range 400–720 nm sampled at 10 nm intervals. To reduce non-imaging noise in the unaveraged source data and to shorten computational time, the spatial resolution of all images was adjusted to a size of 336 × 256 pixels by spatial averaging for all analyses.^13^

#### Cone excitations

For each scene and illumination condition, the spectrum of the light at each pixel in a hyperspectral image was converted to cone excitations based on the 2-deg Stockman and Sharpe cone fundamentals.^75^^,^^76^ Let *L*(*u*,*v*;*λ*) be a hyperspectral radiance image, indexed by spatial coordinates *u*, *v* and wavelength *λ*. If *S*_L_(λ), *S*_M_(λ), *S*_S_(λ) are the long-, medium-, and short-wavelength-sensitive cone spectral sensitivities, measured at the cornea, that is, incorporating preceptor absorption, then at each point (*u*, *v*) the corresponding cone excitations *q*_L_, *q*_M_, *q*_S_ are given by (Equation 1), (Equation 2), (Equation 3).

(Equation 1) *q*_L_*= ∫ L*(*λ*)*S*_L_(*λ*) d*λ*

(Equation 2) *q*_M_*= ∫ L*(*λ*)*S*_M_(*λ*) d*λ*

(Equation 3) *q*_S_*= ∫ L*(*λ*)*S*_S_(*λ*) d*λ*

Integrations were performed numerically over the range 400–720 nm with step size 10 nm.

#### Viewing procedure

In both outdoor and indoor measurements, after completing the gaze calibration, each observer freely viewed the scene for 5 min, in the knowledge that they would be asked a randomly chosen question about the contents of the scene (e.g., how many tree trunks were present). The emphasis was on capturing natural behaviors in free viewing, without the influence of task demands such as searching for or detecting a specific target. A fixation recalibration (recentering) was carried out every minute during the viewing period. At the end of the measurement, the gaze calibration was checked, and, if valid, the gaze data were saved. For one observer, there was a small gaze misalignment with two of the seven scenes, which was not improved after repeating the observation, but the data were retained in the analysis.

Experimental procedures complied with current guidelines of the Research Ethics Committee of the University of Minho to the Color Science Laboratory (CEICVS 052/2021).

### Quantification and statistical analysis

#### Analysis of gaze patterns

Figure S2 shows a schematic of the analysis. On the left, the “Observation of a scene” image represents an observer shifting their gaze at hypothetical times *t*_1_ to *t*_4_. On the right, the “Spatial filtering” image depicts the inverted retinal image within a 5°-diameter region on the retina at time *t*_1_. This angular range was chosen to fit within the acceptance angle of the hyperspectral imaging system and corresponds approximately to the size of the fovea.^82^^,^^83^ To accommodate varying cone density with eccentricity, the projected image was convolved with a two-dimensional Gaussian filter with standard deviation 1 pixel at the central fovea, corresponding approximately to 1 arcmin, the minimum angle of resolution. As eccentricity increased away from the central fovea, the standard deviation was increased by the reciprocal of the cone density,^84^ depicted below the circular image. The result is shown in the “Retinal Images” *f*_1_, *f*_2_, *f*_3_, *f*_4_ at times *t*_1_, *t*_2_, *t*_3_, *t*_4_, respectively.

Possible systematic differences in spectral diet were tested at the center of the fovea *p*_f_ (white filled circle), which corresponds to a single pixel in the filtered retinal image. The difference between local and global adaptation was evaluated using the entire 5°-diameter retinal image. The hyperspectral image was padded with its own mirror image to prevent edge discontinuities and to avoid the central test location sampling more points than the other locations. The imaged area of the original scene was thus assumed to be characteristic of the unimaged areas.

#### Analysis of history of cone excitations at fixation point

The relationship between local and global photoreceptor sampling was quantified by the correlation between local and global histograms of cone excitations, estimated as described earlier. Figure S3 illustrates the time-course of cone excitations. The square colored patches show RGB renderings of spectra sampled at the center of fovea *p*_f_ for the four specific retinal images *f*_1_, *f*_2_, *f*_3_, and *f*_n_ sampled at times *t*_1_, *t*_2_, *t*_3_, and *t*_n_, respectively. Below are shown corresponding histograms of the logarithm to the base 10 of L, M, S cone excitations accumulated over time from the start of the measurement with the first image frame to a specific end frame *f*_*n*_, where *n* increased from 50 to 15,000 in increments of 1. The upper row of histograms is for local excitations and the lower row for global excitations.

The number of data points in the local histograms depended on the end frame. For instance, the first histogram used 50 data points and the last histogram used 15,000 data points. The bin number was systematically determined by the Freedman-Diaconis rule,^85^ which is robust to outliers. The same bin number was used for global histograms from the entire image. The correlation between the relative frequencies of the global and local histograms was repeatedly obtained for all end frames, resulting in 14,951 product-moment correlation coefficients.

#### Analysis of local retinal adaptation and influence of illumination

The extent to which local retinal adaptation discounts the effects of a global illumination change was quantified colorimetrically. Recall that in normal viewing conditions, retinal adaptation to a change in illumination is rarely complete,^86^^,^^87^ and colorimetric models of chromatic adaptation usually incorporate a weighting coefficient, the degree of adaptation *D*, which ranges from 0 to 1.^88^ For simplicity, this factor *D* is omitted from the present calculations.

The local adaptation was estimated at time *t*_*n*_ from a weighted sum of the history of retinal images from the first image frame *f*_1_ until *f*_*n*_. As illustrated by the plot of weight against time in Figure S4, weights decreased exponentially from the largest associated with the most recent image. The estimated adaptation was applied pixel-wise in a diagonal matrix transformation to the retinal image sampled at the subsequent time *t*_*n*+1_. Because of computational demands, six discrete times were used with roughly equal logarithmic steps from *t*_3_ to *t*_14,999_, where *t*_3_ was chosen as the start since there is evidence that an exposure of 60 ms can influence subsequently perceived color.^53^ If observers uniformly sampled an image, the expectation would be that all cones would adapt to the scene’s mean color and local adaptation would coincide with global adaptation.

The mean correlated color temperature (CCT) of the direct illumination across all scenes, measured from the reference surface in each scene, was 5,571 K, with a range from 4,421 K to 7,643 K. To provide a controlled change in illuminant, the effective spectral reflectance image of the scene was multiplied pointwise by a daylight illuminant with CCT 4,000 K,^89^ which approaches the lower limit of outdoor illuminant changes^90^ and sufficient here to test chromatic adaptation.

Color differences Δ*E* between images corrected for local and global adaptation were evaluated in an approximately uniform color appearance space CIECAM16-UCS,^44^ which uses the CIE 1931 colorimetric system rather than those of Stockman and Sharpe.^75^^,^^76^ This space is more uniform than CIELAB color space,^34^ also used in similar analyses. Spectral radiances at each point *i*, *j* under a 4,000 K global illuminant were converted to tristimulus values *X*_*i*,*j*_*,Y*_*i*,*j*_*,Z*_*i*,*j*_ and then to cone-like responses *R*_*i*,*j*_*,G*_*i*,*j*_*,B*_*i*,*j*_ defined in CIECAM16-UCS by (Equation 4), (Equation 5):

(Equation 4) $$\left(\begin{array}{c} R_{i,j}\\ G_{i,j}\\ B_{i,j} \end{array}\right)=M_{16}\left(\begin{array}{c} X_{i,j}\\ Y_{i,j}\\ Z_{i,j} \end{array}\right)$$

where

(Equation 5) $$M_{16}=\left(\begin{array}{c} 0.401\\ -0.250\\ -0.00208\; \end{array}\begin{array}{c} 0.650\\ 1.20\\ 0.0490 \end{array}\;\begin{array}{c} -0.0515\\ 0.0459\\ 0.953 \end{array}\right)$$

After local diagonal matrix transformation, the corrected cone excitations are given by Equation 6.

(Equation 6) $$\left(\begin{array}{c} R_{i,j}^{\prime }\\ G_{i,j}^{\prime }\\ B_{i,j}^{\prime } \end{array}\right)=\left(\begin{array}{c} a_{i,j}\\ 0\\ 0 \end{array}\;\begin{array}{c} 0\\ b_{i,j}\\ 0 \end{array}\;\begin{array}{c} 0\\ 0\\ c_{i,j} \end{array}\right)\left(\begin{array}{c} R_{i,j}\\ G_{i,j}\\ B_{i,j} \end{array}\right)$$

where the scalars *a*_*i,j*_*, b*_*i,j*_*, c*_*i,j*_ depend on spatial position *i*, *j* and are set by the reciprocal values of *R*_*i*,*j*_, *G*_*i*,*j*_, *B*_*i*,*j*_ in the locally adapted retinal image. Analogously, after the global diagonal matrix transformation, the corrected cone excitations are given by Equation 7

(Equation 7) $$\left(\begin{array}{c} R_{i,j}^{\prime \prime }\\ G_{i,j}^{\prime \prime }\\ B_{i,j}^{\prime \prime } \end{array}\right)=\left(\begin{array}{c} a\\ 0\\ 0 \end{array}\;\begin{array}{c} 0\\ b\\ 0 \end{array}\;\begin{array}{c} 0\\ 0\\ c \end{array}\right)\;\left(\begin{array}{c} R_{i.j}\\ G_{i.j}\\ B_{i.j} \end{array}\right)$$

where the scalars *a*, *b*, *c* are constants that depend only on the global spatial means (i.e., $\bar{R}\text{.}$, $\bar{G}\text{.}$, $\bar{B}\text{.}$). Note that the automatic chromatic adaptation stage in CIECAM16 was omitted to avoid double adaptation.

Figure S4 illustrates a hypothetical sequence where the observer shifts gaze at time *t*_1_ to time *t*_5_ (top image), where *t*_4_ is the current time and *t*_5_ is the time of the next frame, 20 ms after *t*_4_. Estimates for two time constants, 1 s and 10 s, are shown, which fall within the range reported previously.^53^^,^^86^^,^^91^^,^^92^^,^^93^ Local and global diagonal matrix transformations were applied to discount the illuminant influence (bottom images).

As shown at the bottom of the figure, color differences Δ*E*_*i*,*j*_ between the local and global images were calculated at each point *i*, *j*, and the median was taken to avoid the influence of outliers. A value of zero at every point indicates the same cone adaptation locally and globally and a positive value indicates spatial inhomogeneity. Although gaze patterns were not recorded with images of scenes under a 4,000 K illuminant, it was assumed that gaze patterns were the same as with the original images.

#### Statistical analysis

To test the effect of the time constant on the minimum Δ*E* (averaged across scenes) in Figure 6, we conducted a one-way repeated-measures ANOVA across the seven scenes using the anova function in MATLAB’s Statistics and Machine Learning Toolbox.
