# Supplementary material for: Eye movements efficiently expose single cone photoreceptors to global scene color statistics
Source: iScience. 2026 Feb 9;29(3):114948. doi: 10.1016/j.isci.2026.114948 (PMC12955238; doi:10.1016/j.isci.2026.114948)
Supplement: Document S1. Figures S1–S4 [file mmc1.pdf]

iScience, Volume *29*

Supplemental information

Eye movements efficiently expose single cone photoreceptors to global scene color statistics

Takuma Morimoto, Luna Wang, Kinjiro Amano, David H. Foster, and Sérgio M.C. Nascimento

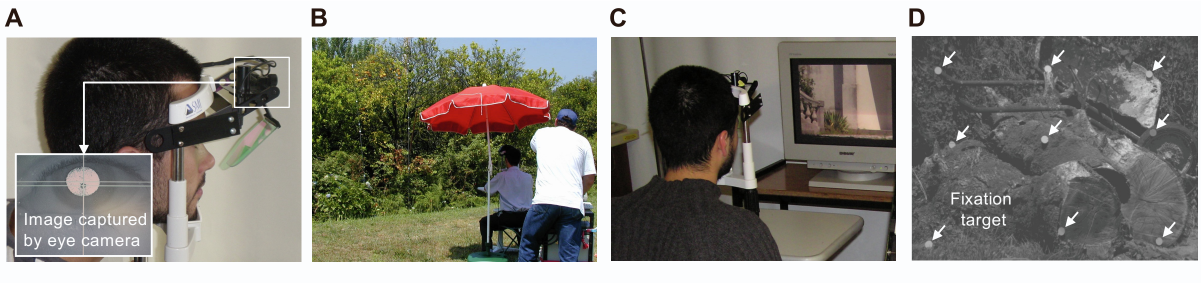

Figure S1. Eye-tracking setup, gaze recording in outdoor and indoor environments, and calibration procedure. (A) The system used for the measurement of gaze position. (B) Gaze recording outdoors. (C) Gaze recording in the indoor laboratory. (D) Nine fixation targets placed in a scene to calibrate the eye tracker.

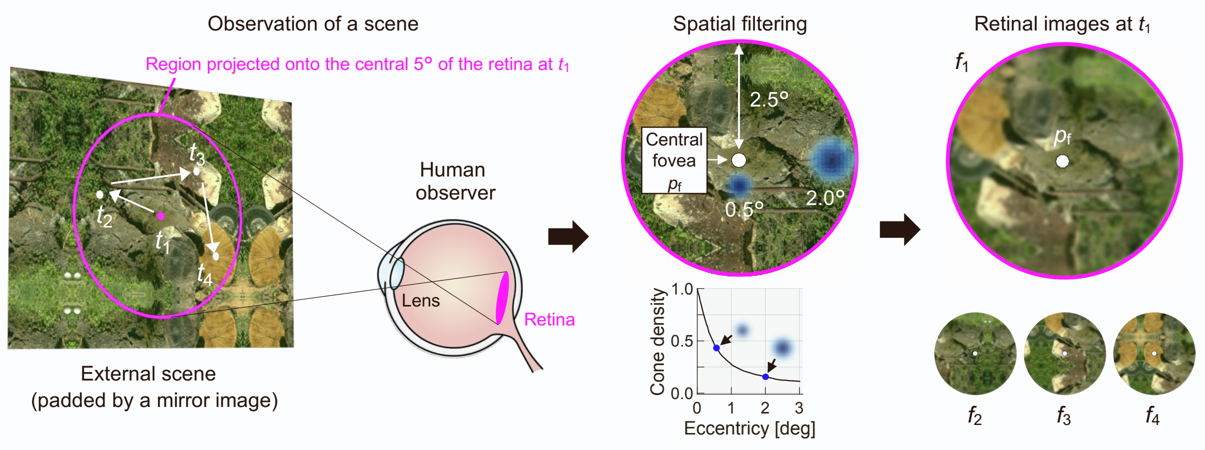

Figure S2. Schematics of the simulation and analysis using recorded gaze data. At a given time point t_1 when the observer is fixating on the center of the scene indicated by the small magenta dot, a circular portion of the scene is projected onto the observer's retina. Spatial filtering to account for cone density variation with eccentricity was applied using a 2D Gaussian, whose standard deviation was determined by the cone density at each corresponding eccentricity. This produces retinal images f_1, f_2, f_3 and f_4 sampled at time t_1, t_2, t_3, and t_4, respectively.

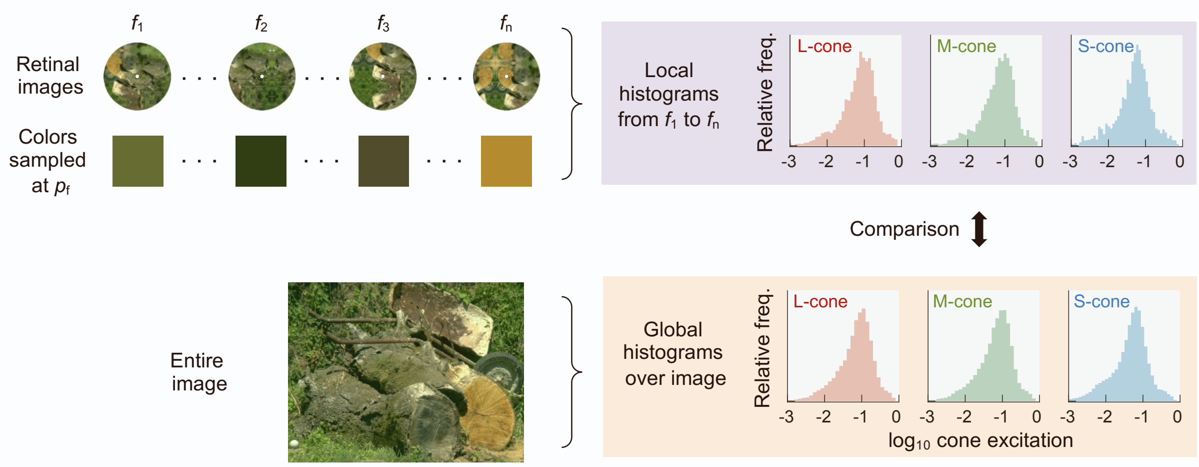

Figure S3. Time-course of excitations of individual cones at the center of gaze. The four circular images on the top left display retinal images f_1, f_2, f_3 and f_n, with RGB renderings of spectra sampled at p_f shown below. The top-right histograms represent local excitations accumulated from f_1 to f_n at p_f. The bottom row shows the global histogram drawn from the entire image.

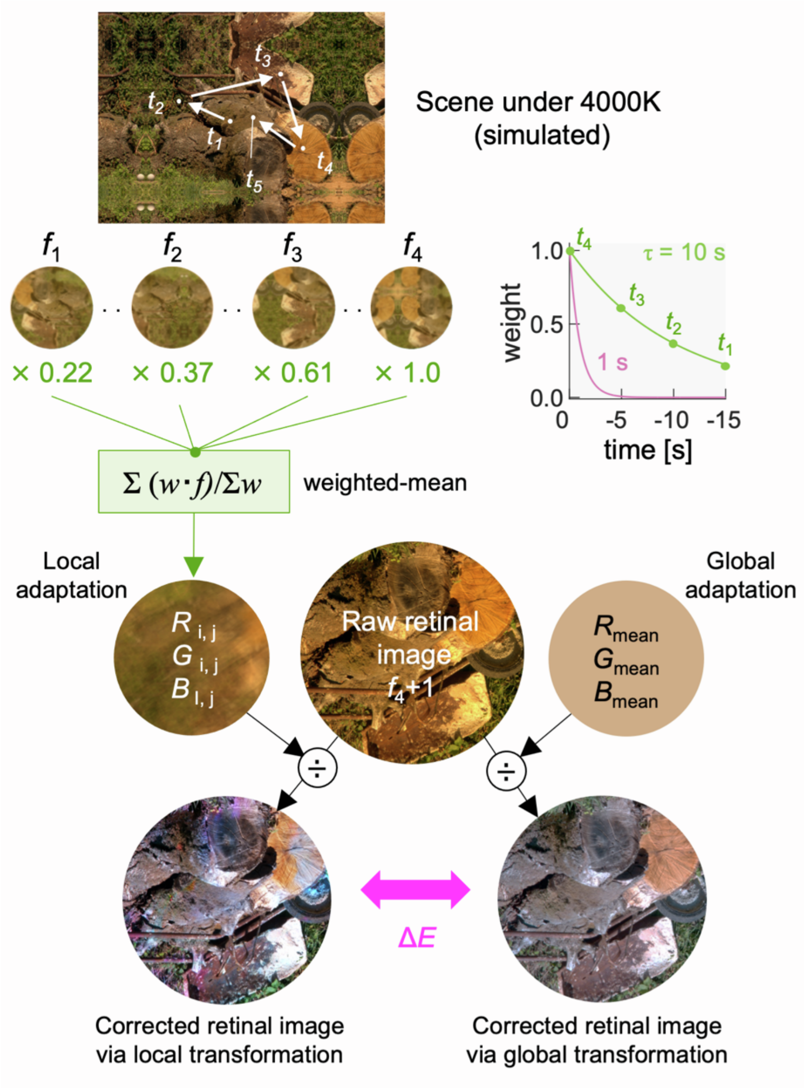

Figure S4. Local adaptation and discounting a simulated global illuminant. Local adaptation was estimated as a weighted sum of past retinal images, with time constants of 1 and 10 seconds considered. In contrast, global adaptation was based on the mean color of the entire scene. After illuminant correction, color differences Δ*E* between images corrected for local and global adaptation were evaluated. RGB refers to 'cone-like' responses defined in the CIECAM16 color space.
